# *Wii Fit U* intensity and enjoyment in adults

**DOI:** 10.1186/1756-0500-7-567

**Published:** 2014-08-26

**Authors:** Julien Tripette, Haruka Murakami, Takafumi Ando, Ryoko Kawakami, Noriko Tanaka, Shigeho Tanaka, Motohiko Miyachi

**Affiliations:** National Institute of Health and Nutrition, 1-23-1 Toyama, Shinjuku, Tokyo, 162-8636 Japan

**Keywords:** Active video game, Exergame, *Wii Fit*, Indirect calorimetry, Metabolic chamber, Enjoyment, Physical activity

## Abstract

**Background:**

The *Wii Fit* series (Nintendo Inc., Japan) provides active video games (AVGs) to help adults to maintain a sufficient level of daily physical activity (PA). The second generation of home AVG consoles is now emerging with new game modalities (including a portable mini screen in the case of the new *Wii U*). The present study was performed to investigate the intensity and enjoyment of *Wii Fit U* games among adults.

**Findings:**

Metabolic equivalent (METs, i.e., intensity) of the *Wii Fit U* activities were evaluated using metabolic chambers in 16 sedentary adults (8 women and 8 men). A short version of the physical activity enjoyment scale was completed for each activity. *Wii Fit U* activities were distributed over a range from 2.2  ±  0.4 METs (Hula dance) to 4.7  ±  1.2 (Hip-hop dance). Seven activities were classified as light-intensity PA (<3 METs) and 11 activities as moderate-intensity PA (3 – 6 METs). The new portable mini screen game modality does not induce higher METs. Men exercised at higher intensities than women. There was no correlation between enjoyment and MET values in women or men.

**Conclusions:**

More and more moderate-intensity activities are available through video gaming, but the average intensity (3.2  ±  0.6) is still low. Users should be aware that AVGs alone cannot fulfill the recommendations for PA, and the video games industry still must innovate further to enhance gaming intensity and make the tool more attractive to health and fitness professionals.

## Findings

### Background

Home-based active video games (AVGs) have been presented in recent years as a potential leisure physical activity (PA) [1]. Several single-session studies classified AVGs as light-to-moderate PA [2], which may not be sufficient to reach current recommendations for daily moderate-to-vigorous physical activity (MVPA) [3]. Although intervention studies have failed to increase the total amounts of PA in children and adolescents [4, 5], there have been reports of beneficial effects of AVGs with regard to body composition outcomes, especially in the adult population [6–9]. As there is no consensus regarding the use of AVGs as a means to increase daily PA, and because the video game industry is still developing rapidly, data on AVG intensity and enjoyment must be frequently updated to help health and fitness professionals to include AVGs in exercise rehabilitation programs.

The popular *Wii Fit* series (Nintendo Inc., Japan) provides AVGs designed to help adults to maintain a sufficient level of daily PA. While the second generation of home AVG consoles is now emerging, the new *Wii U* system includes an independent portable monitor allowing a 360° motion game modality that may increase player movement, upper limb activation, and subsequent energy expenditure (EE). The present study was performed to investigate the intensity of each new *Wii Fit U* activity in adults. A standardized protocol using metabolic chambers (i.e., similar to that used for testing the activities in the previous *Wii Fit series* [10]) was used to facilitate data interpretation and comparison. Heart rate and enjoyment data are also provided.

## Methods

Sixteen Japanese men (*n*  =  8) and women (*n*  =  8) aged between 30 and 45 years old participated in this study. According to Nintendo commercials and packaging materials, sedentary young and middle age adults have indeed been identified as the main target audience for the *Wii Fit series*. The subjects did not report any exercise habits (here defined as at least 30 min of structured physical exercise twice per week during the previous year), and are consequently referred as sedentary elsewhere in this manuscript. Subjects had limited experience with *Wii Fit* (no experience with the tested activities) and were therefore considered as beginners. No training sessions were scheduled before the experimental day. Subjects did not report any chronic diseases.

The standardized metabolic chamber protocol was described in detail elsewhere [10]. Briefly, subjects performed the 18 new activities of *Wii Fit U* at the beginner level. Each activity was continued for 8 min to obtain steady-state EE. Two open-circuit indirect metabolic chambers (15000 or 20000 L) were used to measure the metabolic equivalents for each activity (METs) as an index of intensity. Chambers were equipped with a TV, a video game console, a table, and a chair. Temperature was controlled at 25°C and relative humidity was set at 55%. The oxygen and carbon dioxide concentrations of the air supply and exhaust were measured by mass spectrometry (ARCO-1000A-CH; Arco System, Japan). The flow rate exhausted from the chamber was measured by pneumotachography (FLB1; Arco System, Japan). The oxygen consumption and carbon dioxide production were determined from the flow rate of exhausted air and the concentrations of the inlet and outlet air of the chamber. Data were recorded every 12 s. EE was determined using Weir’s equation [9]. Finally, MET values were calculated from resting EE and steady-state EE during each *Wii Fit U* activity. Heart rate (HR) was monitored throughout the protocol (BIOVIEW1000A PB1811; NEC Medical Systems, Japan).

Finally, a short version of the physical activity enjoyment scale (sPACES) was completed after each activity. Five items from the original 18-item test were chosen. The same items had been used previously by Graves et al. (2010) in a similar protocol [11]. Each item is presented as a 7-point Likert-type scale. The responses for the 5 items were summed to give a score ranging from 5 to 35, and the percentage enjoyment score was then calculated [11].

**Figure 1:**
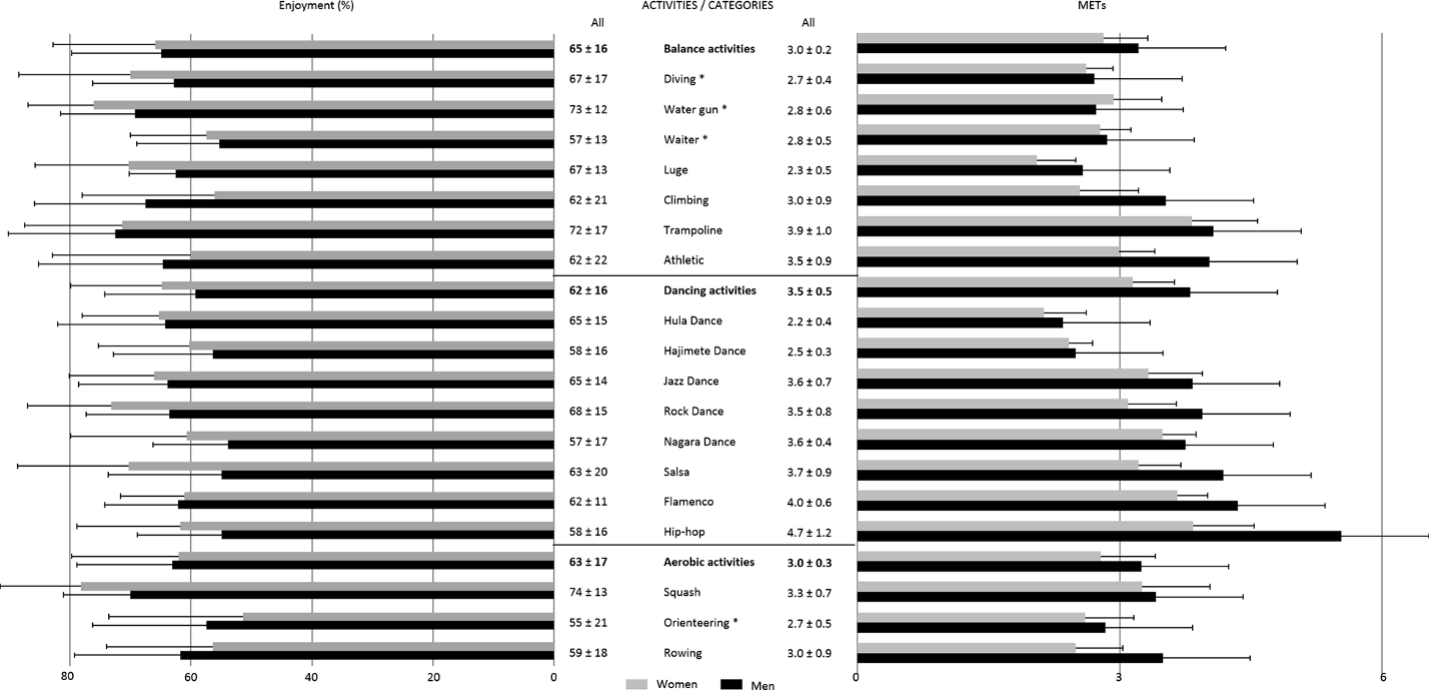
**MET values and enjoyment in the 18 new** ***Wii Fit*** **series activities.** *: Activities that use the portable monitor game modalities.

Statistical analysis was conducted at three levels:

1. For each activity: For intensity and enjoyment, the differences between activities and sexes were tested by two-way ANOVA with 18 repeated measures.
2. For each activity category: In accordance with the manufacturer’s label, activities were classified into three categories (Figure 1): balance activities *vs.* dance activities *vs.* aerobics activities. The differences between these three categories were investigated by one-way ANOVA. The Student–Newman–Keuls test was used for post hoc analysis.

3) For each game modality: Activities were assigned to one of the following modalities (2 modalities, see asterisk in Figure 1): new portable mini screen *vs.* other traditional *Wii Fit* game modalities (*Wii remote* and/or *Wii balance board*). The difference between the two modalities was tested by unpaired *t* test.

In addition, the correlations between intensity and enjoyment scores were investigated for women and men by the Pearson correlation test. METs values and enjoyment scores are shown in Figure 1 as means  ±  SD. Significance is shown as *P*  <  0.05 or *P*  <  0.001.

The protocol was approved by the Ethics Committee of the *National Institute of Health and Nutrition* and subjects gave their informed consent.

## Results

The baseline characteristics of subjects are presented in the Table 1.

**Table 1:** **Subject baseline characteristics**

	Females (***n*** = 8)	Males (***n*** = 8)
Age (years)	35 ± 2	38 ± 7
BMI (kg · m^-2^)	19.1 ± 1.8	25.0 ± 3.6
Resting metabolic rate (kcal · kg^-1^ · h^-1^)	0.88 ± 0.08	1.11 ± 0.20
Resting heart rate (bpm)	72 ± 9	70 ± 7

According to the repeated measures test, intensities were significantly different between activities (*P*  <  0.001). As indicated in Figure 1, the new *Wii Fit U* activities were distributed over a range from 2.2  ±  0.4 (Hula dance) to 4.7  ±  1.2 (Hip-hop dance) METs. Seven activities were classified as light-intensity PA (<3 METs) and 11 activities as moderate-intensity PA (3 – 6 METs). No activity was considered as vigorous PA (>6 METs). The average intensity was significantly higher in men than in women (3.5  ±  0.8 *vs*. 3.0  ±  0.5 METs, respectively, *P*  <  0.001; overall average: 3.2  ±  0.6). HR varied from 86  ±  14 bpm (Hula dance) to 109  ±  16 bpm (Hip-hop dance).

Dancing activities (3.5  ±  0.5 METs) were significantly more intense than balance (3.0  ±  0.2 METs, *P*  <  0.05) or aerobic activities (3.0  ±  0.3 METs, *P*  <  0.05). Finally, the activities with the new portable mini screen game modality were significantly less intense than those with the *Wii remote* or *Wii balance board* only (2.8  ±  0.5 *vs.* 3.4  ±  0.7 METs, respectively, *P*  <  0.001). These activities were rated as light-intensity PA (see asterisk in Figure 1).

Enjoyment scores were found to be relatively homogenous between activities, with the enjoyment scale varying from 55%  ±  21% to 74%  ±  13%. There were no significant differences between activities or sexes. Finally, no relations were found between intensity and enjoyment in women (R  =  –0.01) or men (R  =  –0.07).

## Discussion

The new *Wii Fit U* game provides an extension of slightly higher intensity activities (2.2  ±  0.4 to 4.7  ±  1.2 METs, mean: 3.2  ±  0.6 METs) in comparison with activities included in the previous *Wii Fit +* (1.3  ±  0.4 to 5.6  ±  1.1 METs, mean: 2.5  ±  1.0 METs, see [10]). While the new portable mini screen did not result in higher intensity activity in the present study (mean: 2.8  ±  0.5 METs), more MVPAs are still available through activities that use the traditional *Wii* game modalities (i.e., the *Wii balance board* and/or *Wii remote*). Surprisingly, the dance activities were found to be more intense than the aerobic activities (3.5  ±  0.5 *vs.* 3.0  ±  0.3 METs, respectively). One reason is that most of the dances included in the *Wii Fit U* package are actually practiced as dance aerobic exercises. Another reason is that the experiment was performed in beginner mode, which could have significantly lowered the intensity of the activities, including aerobics activities. These observations emphasize the necessity for health and fitness professionals not to rely on the manufacturer’s labeling, as the terminology does not necessarily reflect the effective intensities of the activities and may be misleading.

Attempting to increase PA in adults is a huge challenge that requires multifaceted strategies, and the present results may support the inclusion of AVGs in such strategies. In the present study, adult men expended more energy than women playing the same video game. However, this result cannot be explained by a difference in enjoyment as there was no difference in sPACES score between the sexes. Finally, no relation was found between enjoyment and intensity, which could be a critical point to maintain subject adherence over longer periods. Both the scientific field (*via* longitudinal studies) and the video game industry should address this important issue.

Significant proportions of *Wii Fit U* activities are still rated as light-intensity PA (7 of the 18 new *Wii Fit U* activities tested; 53 of 86 overall, see [10]). While these activities cannot be relied upon to fulfill current recommendations for PA (that should be MVPAs) [3], they may still be valuable to break sedentary times, or for seniors who would have greater benefits from light-intensity PA compared to their younger counterparts [12]. However, further studies specifically designed to address these points are required.

### Limitations and strengths

In the present study, all of the participants were invited to play at the beginner level, which may have lowered the game intensities. Indeed, Worley et al. [13] reported that playing *Wii Fit* games at an intermediate level significantly increased players’ EE. Another limitation is that the whole study was conducted in single-player mode. Multi-player modes, which may better reflect some real-life situations, have indeed been found to be more intense [14–16]. However, the use of the single-player mode allows a fairly objective evaluation of each game intensity, providing important information to the players and health and fitness professionals who wish to select AVGs for their usage. On the other hand, in real-life situations, adult players are invited to play games with their relatives and at intermediate to expert levels to have greater benefits from their AVG sessions. Finally, the relative small sample size, population age, and characteristics (i.e., inexperienced), as well as the 8-min format chosen for each activity, may prevent generalization of our results. However, one strength of the present study was the use of the same standardized protocol as described previously [10], allowing study-to-study comparisons, thus providing information about the evolution of *Wii Fit series* intensity.

## Conclusions

While the new *Wii Fit U* tends toward more moderate-to-vigorous physical activity, the average intensity is still low (3.2  ±  0.6 METs). Users (including both players and health and fitness professionals) should be aware that active video games cannot be relied upon to entirely fulfill the recommendations for physical activity. Consequently, recommendations for sedentary people to engage in exercise should only be made for some selected games and must be considered as only one tool in a wider multifaceted strategy. On the other hand, innovative game modalities allowing more vigorous exertion could be developed to break the light-intensity physical activity/moderate-to-vigorous physical activity barrier more significantly and make these games more attractive for the purposes of health promotion.
